# What Is the Heart? Anatomy, Function, Pathophysiology, and Misconceptions

**DOI:** 10.3390/jcdd5020033

**Published:** 2018-06-04

**Authors:** Gerald D. Buckberg, Navin C. Nanda, Christopher Nguyen, Mladen J. Kocica

**Affiliations:** 1 Department of Cardiothoracic Surgery, David Geffen School of Medicine at UCLA, 10833 Le Conte Avenue, Room 62-258 CHS, P.O. Box 951741, Los Angeles, CA 90095-1741, USA; 2 Division of Cardiovascular Diseases, University of Alabama at Birmingham, 1900 University Boulevard, Birmingham, AL 35233, USA; nanda@uab.edu; 3 Cedars-Sinai Medical Center, 8700 Beverly Blvd. PACT, Suite 800, Los Angeles, CA 90048, USA; christopher.nguyen@cshs.org; 4 UC Clinical Centre of Serbia, Clinic for Cardiac Surgery, 8th Kosta Todorovic St., 11000 Belgrade, Serbia

**Keywords:** conventional heart anatomy, helical ventricular myocardial band, mitral valve opening, isovolumic relaxation time, RV function, diastolic dysfunction

## Abstract

Cardiac dynamics are traditionally linked to a left ventricle, right ventricle, and septum morphology, a topography that differs from the heart’s five-century-old anatomic description of containing a helix and circumferential wrap architectural configuration. Torrent Guasp’s helical ventricular myocardial band (HVMB) defines this anatomy and its structure, and explains why the heart’s six dynamic actions of narrowing, shortening, lengthening, widening, twisting, and uncoiling happen. The described structural findings will raise questions about deductions guiding “accepted cardiac mechanics”, and their functional aspects will challenge and overturn them. These suppositions include the LV, RV, and septum description, timing of mitral valve opening, isovolumic relaxation period, reasons for torsion/twisting, untwisting, reasons for longitudinal and circumferential strain, echocardiographic sub segmentation, resynchronization, RV function dynamics, diastolic dysfunction’s cause, and unrecognized septum impairment. Torrent Guasp’s revolutionary contributions may alter future understanding of the diagnosis and treatment of cardiac disease.

## 1. Introduction

The current approach to understanding cardiac dynamics relies upon movements that adhere to the conventional topographical separation of cardiac muscle into the left ventricle, right ventricle, and septum. Functional analyses have addressed them independently, and this approach has resulted in many suppositions that this report will define and question.

Alternatively, cardiac muscle mass is formed by the helix and surrounding circumferential wrap described by Lower in the 1600s [1], Senac in the 1700s [2], Krehl in the 1800s [3], Mall in the 1900s [4], and more recently by Torrent Guasp [5]. The integrated function of this wrap and helical architectural configuration explains the heart’s mechanical actions [6,7,8].

**Figure 1:**
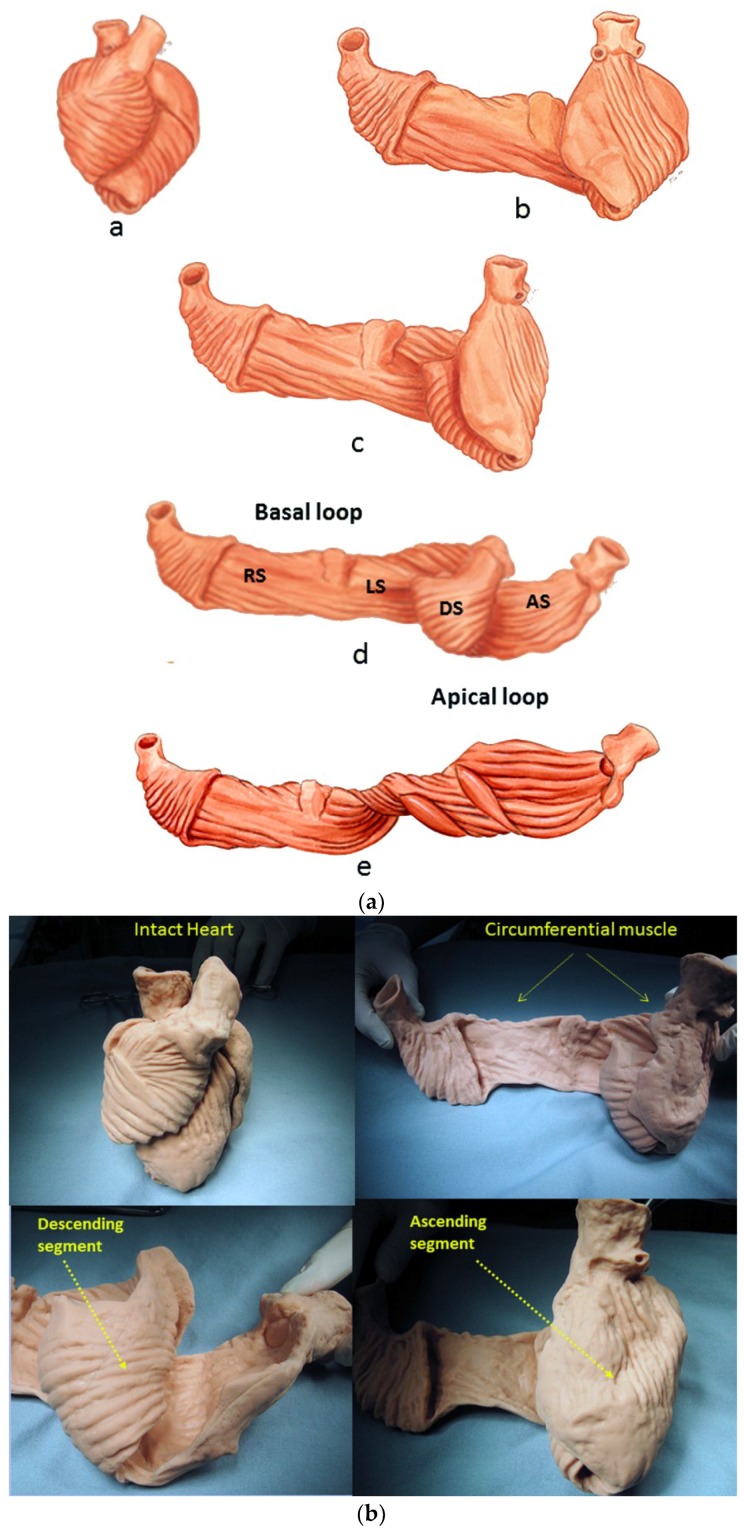
(**a**) Helical ventricular myocardial band unfolding. Upper left—intact heart. Upper right—circumferential or basal loop unfolding its right segment. Second layer—further circumferential or basal loop unfolding of its left segment and showing inner helix. Third layer—helix unfolding to display descending segment (DS) after ascending segment (AS) is separated. The entire basal loop (containing RS and LS) is also shown. Bottom layer—HMVB unraveled to display its rope-like model appearance. Longitudinal fibers only exist within the two papillary muscles; (**b**) unfolding of HVMB model. Upper left—intact heart. Upper right—circumferential wrap or basal loop with transverse fibers and the inner helix. Lower right—unfolded helix showing the oblique fibers of the inner descending helical arm that is separated from the outer ascending helical arm. Lower right—outer ascending helical arm. Marker arrow points to the left anterior descending artery pathway that bisects the helical muscles forming septum and left ventricular free wall. Reproduced form the references [6,8] with Publisher’s permission.

For example, the left ventricular free wall and septum are usually discussed separately, yet both are formed by the same muscle (Figure 1) and their function cannot be separated unless isolated focal lesions exist. For this reason, the anterior descending and posterior descending coronary arteries are simply vascular highways perched upon the top or bottom of the helical muscle forming the septum and its adjacent LV free wall.

Treating disease requires restoration of normality, so decisions must be based upon an understanding of anatomic normality. The heart’s functional counterpart involves only six movements; narrowing, shortening, lengthening, widening, twisting, and uncoiling. The helical ventricular myocardial band model of Torrent Guasp [5] appears in the two classical anatomy texts of Clemente [9,10], and Moore and Dally [11] and its mechanics explain each motion [6,8,12].

This knowledge answers the query about the heart by explaining the only valid definition of heart structure involves describing a mechanical architecture whose motion can account for all dynamic cardiac movements. Misconceptions happen without it. For example, rather than focusing upon cardiac compression, the pivotal role of twisting for mechanical proficiency must be understood [13]. Twisting occurs because the helical cardiac design allows the natural 15% muscle fiber shortening to create a 60% ejection fraction [13], but this truth is not generally appreciated.

The background behind this misunderstanding started 2200 years ago when Erasistratus (280 BC), then Galen (180 AD) and subsequently Borelli (1600s) [14] described twisting, which causes blood to have an ebb and flow motion. But William Harvey, who uncovered the circulation in 1628, also introduced a compression and dilating action (mimicking a clenched and open fist) [15], an action that changed Galen’s ebb and flow hypothesis. His supporters deemed Galen’s concept incorrect, and Harvey’s approach has prevailed for 400 years—gaining further support from two-dimensional imaging (ventriculogram and echo) studies. The newer three-dimensional imaging tools (MRI and speckle tracking) provide the spatial resolution that allowed re-emergence of the clockwise and counterclockwise twisting rotations Erasistratus described 2200 years ago.

The capacity to understand the dynamics of the surrounding wrap and helix is a very different approach from using deductions to explain many ‘accepted cardiac mechanical relationships’. This tactic will lead to questioning of many ‘state of the art’ concepts. They include heart anatomy as LV, RV, and septum, timing of mitral valve opening, the isovolumic relaxation period, structural reasons for torsion/twisting, the term untwisting, structural reasons for circumferential and longitudinal strain, echocardiographic cardiac sub segmentation, resynchronization, RV function dynamics, and diastolic dysfunction’s cause and its unrecognized septum involvement.

## 2. Topographical versus Structural Heart

**Figure 2:**
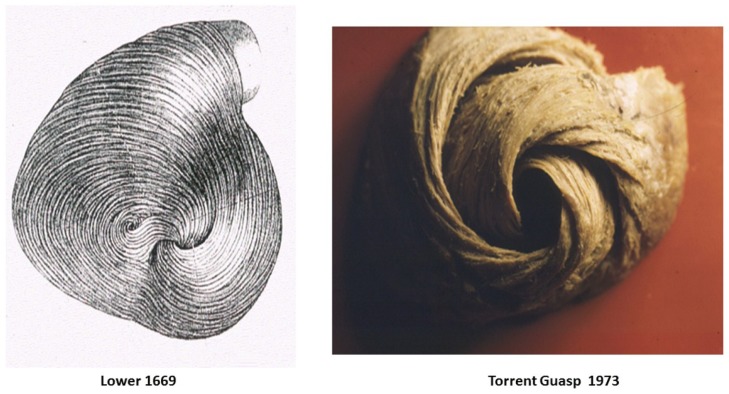
Apical view of heart muscle showing the fibers clockwise and counterclockwise spiral formation. Images display common anatomy from Lower in 1669 (**left**), and Torrent Guasp in 1970 (**right**). Reproduced form the reference [17] with Publisher’s permission.

A heart with two ventricles, separated by a midline muscular septum defines its classic morphologic description. Despite correct topography, no functional insight is provided to define what these three structures do. A different structural guideline has existed for 500 years, whereby heart architecture contains a helix formed by right- and left-handed fibers and a surrounding circumferential wrap [1,2,3,5,16]. This anatomy preceded Torrent Guasp’s identifying the interweaving architecture by unraveling its muscle bundle formation to solve the Gordian Knot of anatomy [5] (Figure 2). His novel description of a helical ventricular myocardial band (HVMB) [17] identifies a vortex at the tip of the apex, which is formed by the overlapping of coiled helical arms. Yet when uncoiled, the entire heart’s rope-like configuration becomes revealed (Supplementary Video S1).

Torrent Guasp’s macroscopic anatomic pattern (Figure 1), whereby the transverse muscle at the base of the heart (circumferential wrap) turns downward to form an inner descending arm of the helix, which creates an apex by turning upward to form an outer ascending helical arm that ends at the aortic root. Grant [18], Lev and Simkins [19], and Anderson [20] questioned his macroscopic findings because of concerns about the validity of his dissection planes in cadaver tissue. Our analysis of the 1971 functional studies by Armour and Randall [21] also questioned the proper site for posterior papillary muscle, but their correct location is now displayed in subsequent HVMB dissections. The only basic element needed to uncover the mechanical reasons for the living heart motions is a valid structure/function relationship comprehension.

**Figure 3:**
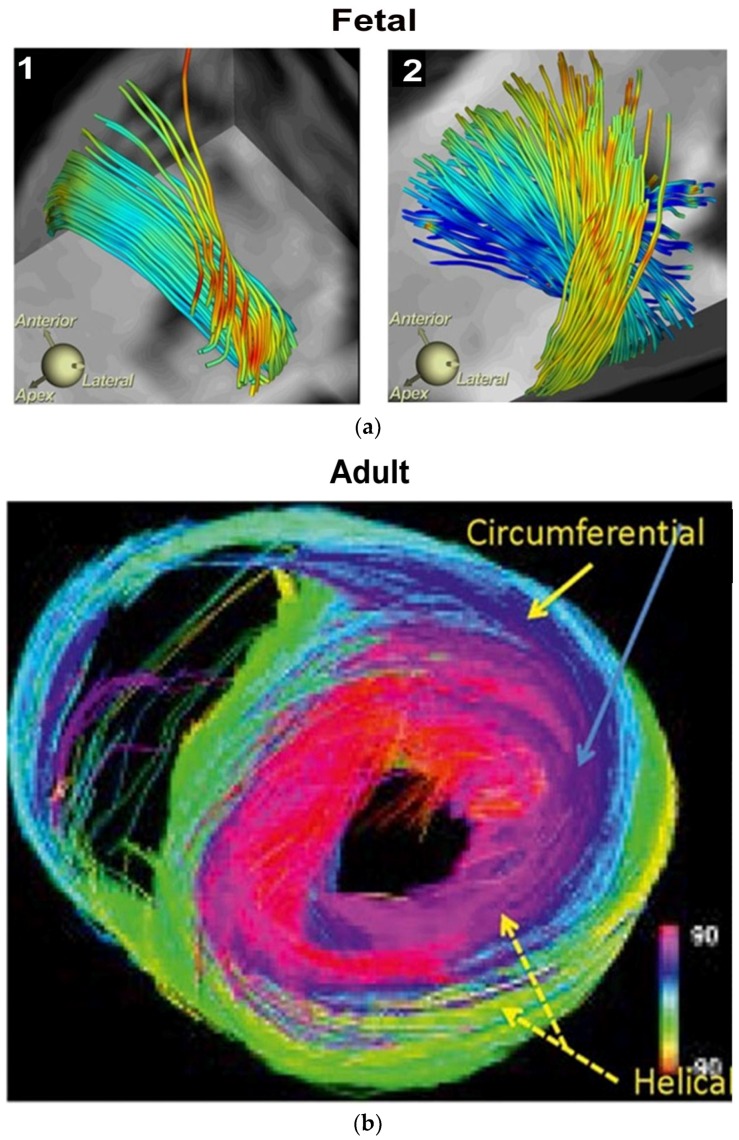
Human myocardial fibrillogenesis: (**a**) 1 and 2 show DT-MRI during the myocardial fibrillogenesis (gradual increase in number and spatial helical arrangement) of the ventricular myocardial fibers in human embryo at 10 and 14 weeks, respectively; (**b**) DT-MRI in adult heart showing helical right handed helix (red), left handed helix (green/yellow) and blue circumferential or horizontal fibers with zero helix. Note absent circumferential fibers in septum. Reproduced form the references [8,24] with Publisher’s permission.

**Figure 4:**
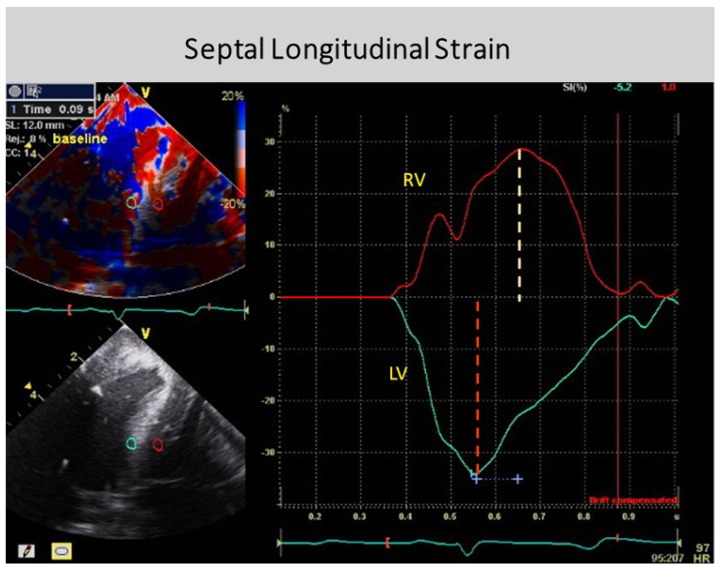
Doppler longitudinal strain imaging of septum (right and left sides) in apical four-chamber view. Longitudinal strain marked by red (right) and green (left) circles, showing deformation in opposite directions on right and left septum sides—relative to baseline zero value. Timing shows LV first and RV second. SR, Strain rate; AVC, aortic valve closure; RV, right ventricle; LV, left ventricle. Reproduced form the reference [38] with Publisher’s permission.

Controversy and Criticism: MacIver, Anderson’s colleague, recently proposed a report to put “an end to unique myocardial band” approach, because its presence cannot be defined by two- dimensional histology [22,23]. Our report is a counterforce to this conclusion, since mechanically explaining cardiac function is the only hallmark for determining the creditability of structure. MacIver questions whether abrupt helical changes occur in a wrapped myocardium by using CT images, thereby taking a position that countermands their presence during gestation, with ongoing helical persistence during adulthood (Figure 3). His argument that a circumferential (horizontal) wrap exists in the septum would functionally erase the twisting motion, which is the most powerful force of cardiac efficiency. It would introduce a wedge that shall impair motion between the gliding helical arms, as shown in Supplementary Video S2 and Figure 4. Moreover, adult DT-MRI images from the same laboratory that generated the prior gestation studies (Figure 3), further documents that circumferential fibers are absent in the septum [24]. MacIver believes thinking twisting is only a minor contributor to stroke volume, thereby presenting a pathophysiology concept that vastly differs from current functional knowledge showing septal twisting is responsible for 80% of right ventricular output [25], and loss of left ventricular twisting is the earliest sign of heart failure [26]. Conversely, the mechanics of the myocardial band completely describes this pathophysiology during health and disease.

Macroscopic analysis has the limitations of not addressing the microscopic display of the nests of layers and lamellae within individual myocytes that interact with cardiac ejection and filling. LeGrice found sarcomeres clustered in 1–12 groups within their connective tissue netting [27,28]. While microscopic relationships are correct, they must also coordinate with the heart’s macroscopic geometric form to fulfill Sallin’s [13] fundamental form/function relationship. This need is emphasized in clinical heart failure where the normal conical heart shape develops a spherical configuration [29]. This geometric change transforms the fiber direction of helical arms into a more transverse architectural fiber orientation which impairs LV systolic and diastolic function [13].

The conceptual understanding of macroscopic cardiac structure is straight forward because only three components need be known. There is a circumferential wrap of transverse fibers that surrounds both ventricles—called a basal loop—which compresses and rotates the global heart, and predominantly forms the RV free wall [25]. The second is the muscular helix that resides within an apical loop nestled within its surrounding circumferential wrap [30]. Its two oblique fiber arms cross each other at 60° angles [31], with the inner helical coil descending from base to apex, and outer helical coil ascending from apex to base. The same helix forms the septum and part of the LV free wall, whose movements are shortening, lengthening and twisting.

Simplicity underlies the solution to the structure function relationship, since its three structural components (the wrap and two helical arms) produce the heart’s six readily apparent dynamic movements of narrowing, shortening, lengthening, widening, twisting, and uncoiling [6,7,8]. This interaction precisely follows Keith’s 1918 Harvean Lecture comment “no theory of function is true unless it explains each detail of structure” [32].

Functional balance exists between the structural wrap and helix, as the wrap causes counterclockwise motion before ejection, the helix produces twisting, and the wrap triggers clockwise recoil during post ejection interval that includes the isovolumic period [6].

State-of-the-art imaging reports do not recognize this dynamic balance because they do not address the fundamental role of the surrounding wrap [33,34,35]. This gap keynotes the hiatus between deduction and anatomic knowledge, especially since this large circumferential wrap muscle mass highlights the framework structural contributions of William Harvey [15], Krehl [3], Mall [4], Robb [16], and Torrent Guasp [5].

Echo motion recordings amplify the circumferential basal loop’s presence by documenting cardiac lengthening during the pre-ejection isovolumic interval [6], while MRI imaging authenticates its global counterclockwise rotation [36,37]. The circumferential wrap’s large muscle mass predominates, and thus overcomes the shortening and clockwise rotation expected from the ongoing subendocardial muscle contraction. A common analogy explains such domination, as one could imagine a train heading south at 60 miles per hour, while its first car houses a runner who speeds northward toward the back car at 6 miles per hour; the power of the southern train always wins, as does the wrap over the helix.

The anatomy documenting the location of the outer circumferential wrap becomes evident from echo studies showing the smooth reciprocal movement (in different directions) between the contracting inner and outer helical arms (Supplementary Video S2). Interposing a transverse band of compressing muscle between these gliding surfaces would interrupt this motion, as well as impair the smooth positive and negative septum strain displacement on longitudinal strain recordings (Figure 4).

**Figure 5:**
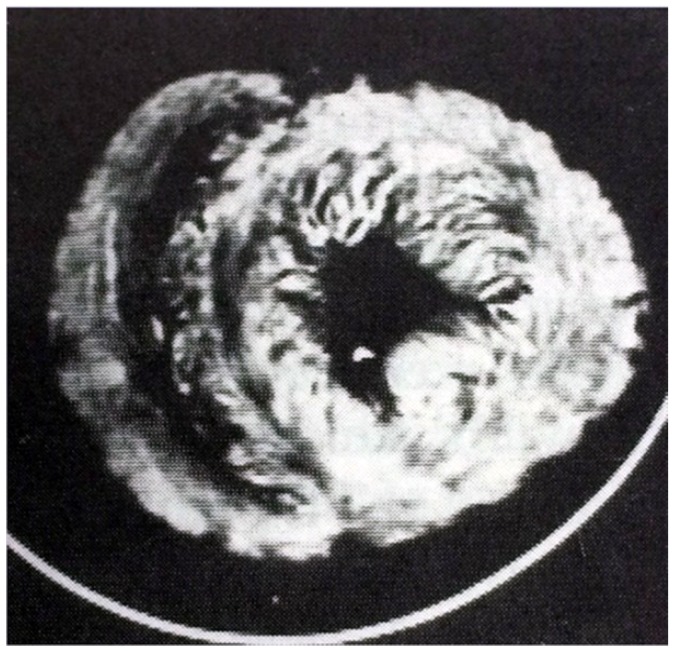
Computerized Ttomography of cardiac short axis, at mid ventricle level, following air insufflation to separate collagen scaffold netting. Plane between the two septum muscle mass rims reflects the echogenic line in septum, and Supplementary Video S4 records motion between these post mortem rims. Reproduced form the reference [39] with Publisher’s permission.

Macroscopic interlocking of structure and function is aided by the comparison of their dynamic clockwise and counterclockwise helical motions in Supplementary Video S2, with their anatomic configuration in post mortem CT images (Figure 5).

**Figure 6:**
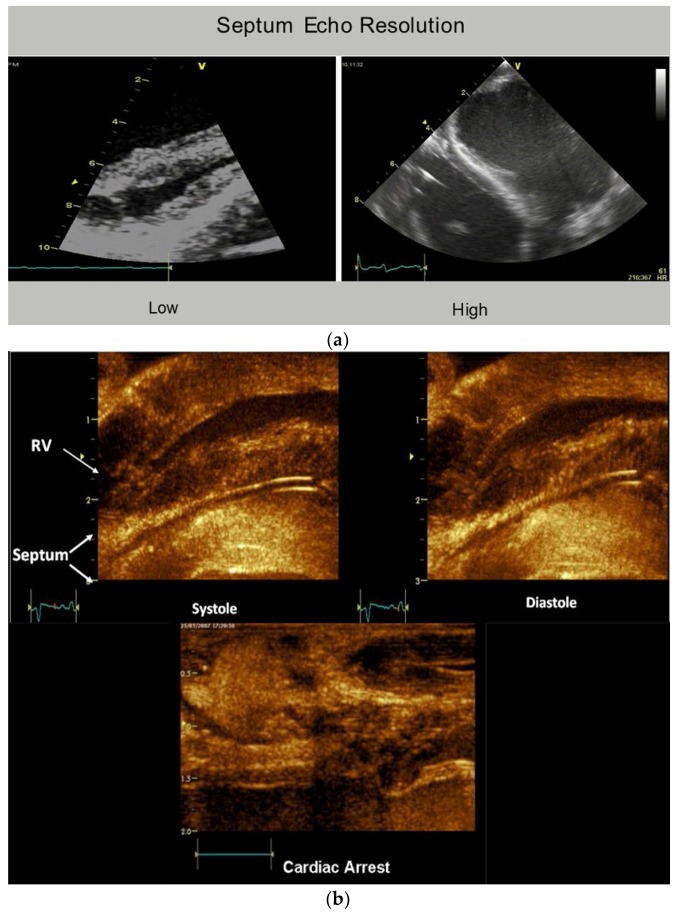
(**a**) Mid-septal hyperechogenic line shown in low and high-resolution echocardiogram; (**b**) septum high-resolution ultrasound image at transducer frequency (12 MHz), displaying its bilayer line with inner dimension structure of 100 to 150 μm. Working septum muscle fibers display different directionality on either side of septum line. This disappears, as does the echogenic line—when cardioplegia (cardiac arrest) stops contractile function. Reproduced form the reference [8] with Publisher’s permission.

The plane identifying the mid ventricular overlapping between helical arms (Supplementary Video S2) extends from apex to base and is called the hyperechogenic zone. It is 1 cm wide in low fidelity tracings, narrows to 3 mm in higher fidelity recordings, and shrinks to 100 μm [12] following higher fidelity echo examination [39,40,41] (Figure 6). The smooth and efficient muscle movements, on either side of it, suggest this zone reflects is a glide path for helical arm motions. The working heart is a precursor for this zone’s presence, since it disappears when cardioplegia arrests the heart [12].

These dynamic findings differ from conclusions made from post mortem DT-MRI observations that suggest circumferential fibers exist within the septum mid myocardium due to observations of zero helix angles [42]. Yet physiologic testing exposes uncertainty about the anatomic validity of DT-MRI findings, because the 100 micron thick echogenic zone disappears following cardioplegia, yet the dead heart septum MRI studies describe a circumferential muscle that occupies half of its muscle mass [43,44].

## 3. How Three Structural Components Cause Normal Functional Dynamics

The most powerful or dominant muscle amongst these three architectural components governs the direction of movement, and their contraction takes place at different timing intervals [45]. For example, the wrap narrows and stretches the ventricle during the pre-ejection isovolumic phase, as it overcomes the simultaneously contracting inner descending helix that should make the ventricle shorten [6,7].

Conversely, shortening during ejection emphasizes the dominance of the inner descending helical arm. The ventricle twists as the base rotates clockwise and apex turns counterclockwise from the torque of the outer ascending helical arm’s longer lever radius [46]. The outer helical coil cannot produce shortening because its longitudinal strain signal is positive (showing elongation) (Figure 4). Despite this, both helical arms exert dominance during twisting because the inner descending coil causes shortening (generating twice the longitudinal strain) [47] while the outer ascending helical coil makes the apex turn counterclockwise [46]. Our imaging tools provide insight into this action via the echogenic septal line (Figure 6) as the echo beam suggests their fiber orientation pathways by passing along or across the ascending and descending muscle masses [12].

**Figure 7:**
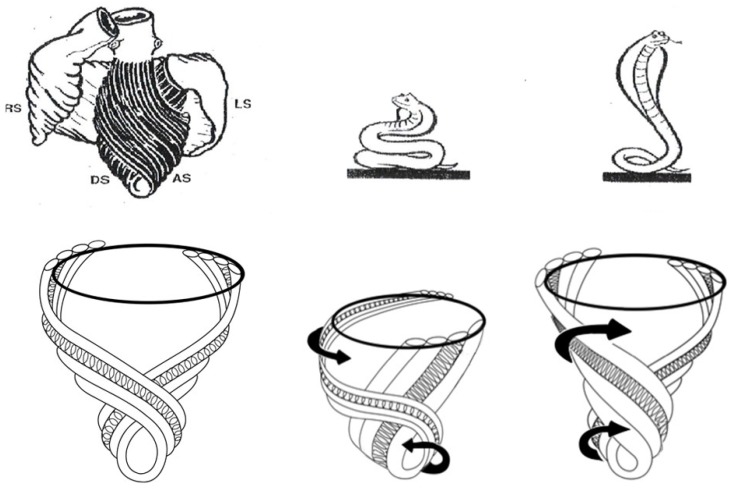
Upper left shows intact heart containing a basal loop (circumferential wrap with right (RS) and left (LS) segments) and helix (dark color) with descending (DS) and ascending (AS) segments. Lower left—diastole without contraction. Lower middle—displays shows torsion (twisting) with stronger contraction of descending helical arm (tighter coils), and stretching of ascending helical arm. Lower right—recoil with global clockwise rotation; note lengthening due to ongoing contraction of ascending helical arm. Upper drawing—cobra shows similar elongation from its contracting muscle. Reproduced form the reference [17] with Publisher’s permission.

The wrap [6,7] causes clockwise global recoil during the post ejection isovolumic phase, as it springs backward to reverse its pre-ejection counterclockwise motion [37,48,49]. The helix is not involved in this component of recoil because its outer ascending arm is still contracting—but the dominant circumferential wrap’s clockwise motion overturns its counterclockwise motion. Simultaneously, the ventricle lengthens from straightening of the contracting outer ascending helical coil that starts when the dominant inner descending helix contraction stops (Figure 7) [7].

Global longitudinal strain measurements match those recorded by sonomicrometry [6] and the observed septum motion in opposite directions reflects the reciprocal fiber orientation of its helical muscles (Figure 4). The outer ascending helical arm starts contracting ~60 ms later than the earlier inner descending helical arm, followed by a ~90 ms timing gap that earmarks the difference between the end of shortening of the inner (descending) arm, followed by the end of outer (ascending) helical arm shortening [45]. This timing hiatus between the end of contraction in the inner and outer helical arms provides a mechanical contradiction (i.e., presence of contraction) to suggestions that repolarization starts earlier in ascending than descending helical arms [50].

## 4. Mitral Valve Opening

**Figure 8:**
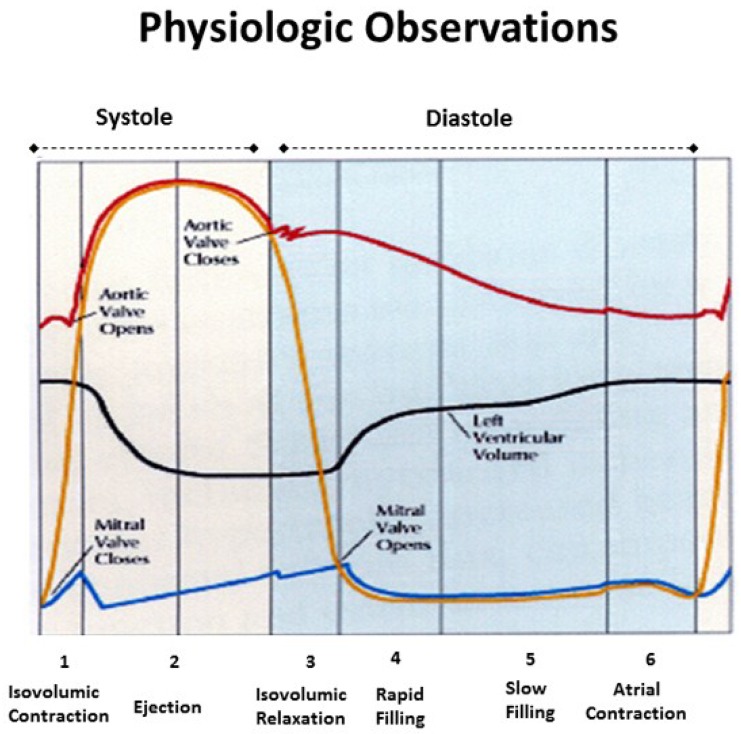
Physiologic observations in all texts and medical journals, showing mitral valve opening (MVO) when LV pressure falls below left atrial pressure, and that the isovolumic relaxation phase exists between aortic valve closure and MVO. Reproduced form the reference [6] with Publisher’s permission.

Mitral valve opening (MVO) is a term reflecting the Doppler based echocardiographic recording of initial transmitral inflow into the left ventricle. This passage coincides with left ventricular pressure falling below atrial pressure, and the most rapid anterior mitral leaflet motion [51]. Its designation is universal and the term MVO appears in text books and journal reports (Figure 8). Yet physical separation of the mitral leaflets is the only valid MVO requirement. Lee in 1990 [52] called MVO “the mitral valve artifact that correlates with the E point in the mitral echogram, but is unrelated to actual mitral valve opening”, and others have also questioned its validity [53,54].

MRI recordings quantify leaflet separation, and Supplementary Video S3 documents that mitral valve leaflets detach from each other at the end of systole—with an open aortic valve. Abrupt loss of leaflet coaptation begins when recoil starts, ~27 ms before aortic valve closure [55], coinciding with negative dP/dt or deceleration of left ventricular pressure. Such MVO documentation exposes an enormous discrepancy between reality versus what has been deduced. The uncoiling process causes MVO, and introduces the muscular action that may trigger diastolic dysfunction.

**Figure 9:**
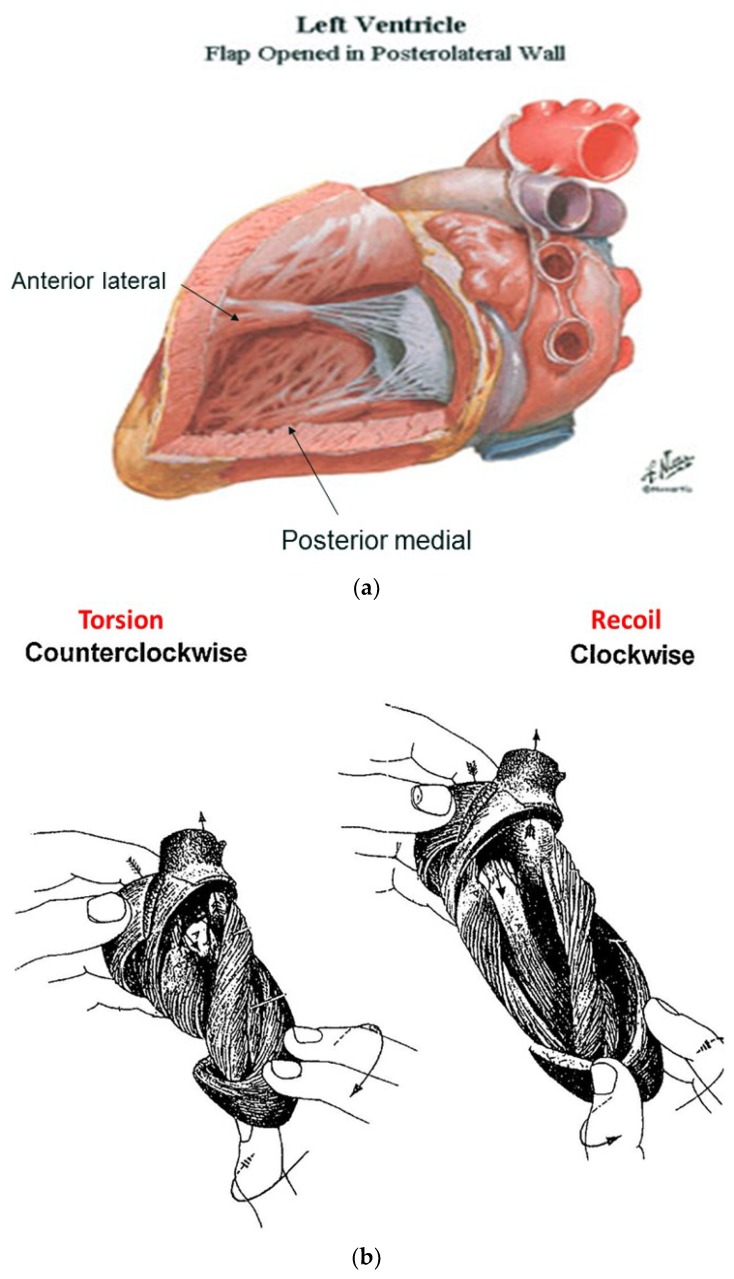
(**a**) Mitral valve apparatus composed of fixed annulus and mobile leaflets, chordae tendineae, papillary muscles, and ventricular wall; (**b**) Mall’s 1911 report showing how apical counterclockwise rotation of the apex shuts the valve during torsion—by bringing spiral papillary muscles together. Mitral valve inflow area opens during from its clockwise rotation during recoil. Reproduced form the reference [56] with Publisher’s permission.

The functional dynamics of the wrap and helix establishes the mechanical insight to explain why MVO occurs during systole. This geometric process focuses upon the architectural interaction between the mobile ventricle that uncoils—and the mitral annulus that is fixed. The circumferential wrap causes clockwise recoiling, which rotates the helix that contains the papillary muscles that connect with the mitral leaflets and annulus. Their counterclockwise rotation during ejection allows them to close the fixed annulus by a leaflet flap, and this ‘trap door’ re-opens—as they spring backward during recoil to produce MVO (Supplementary Video S2). The dynamics of papillary motion are shown in Supplementary Video S4. Figure 9a,b anatomically displays them at rest and during motion. The 1911 report of Mall [4], a renowned anatomist, demonstrates their clockwise and counterclockwise ventricular movements during twisting and uncoiling.

MVO can only happen when helix’s inner descending contraction has ended, an action that permits initiation of outer ascending helix arm lengthening (Supplementary Video S3) as quantified by Karwatowski [57]. These cohesive actions of recoiling (by the wrap) and elongation (by the outer helix arm) reinforce why mechanical factors play such a vital role in mitral leaflet separation.

Our understanding of diastolic dysfunction is enhanced by a fuller understanding of MVO—since uncoiling cannot begin while torsion is ongoing. Ventricular recoil begins at the inception of negative dP/dt, so that this measurement may become a signpost to determine when unwinding starts. If MVO timing is normal, the hiatus or time gap between the end of the inner and outer helix contraction is ~90 ms—and its disruption will impair uncoiling [6,7,58]. Prolonged torsion narrows this gap, so that thinking about the mechanical reasons for MVO may spawn some of the new treatments that will be subsequently considered [56].

Finally, the traditional identification of MVO is incorrect, so that the physiological term “mitral valve inflow” (MVI) should replace it. MVO is due to anatomic changes that carry vast physiologic implications—as we suspect recoiling will become the centerpiece behind understanding diastolic dysfunction.

## 5. Isovolumic Relaxation Time

Isovolumic relaxation time (IVRT) joins MVO in being a universally accepted term (Figure 8). It signifies that part of the cardiac cycle between aortic valve closure and MVO, where the ventricle relaxes (diastole) without lengthening, and ventricular volume is unaltered. Wiggers described it in 1923 [59,60], as does today’s Stedman Medical Dictionary [60].

The ventricle is isovolumic, yet the other three components reflect deductions that are incorrect. First, the MVO during clockwise recoil is caused by the wrap—initiating when LV negative dP/pt starts, rather than when a LV pressure falls below left atrial pressure. Second, the entire ventricle does not relax—ongoing strain measurements and sonomicrometer crystal recordings confirm outer ascending helical arm contraction [6]. Finally, it produces lengthening that is quantified by MRI (Supplementary Video S3), two-dimensional echo [6], and longitudinal strain recordings [38]. This elongation movement may mirror how a cobra develops an erectile stance before striking [17].

Rademakers described the impact of the post ejection isovolumic interval on ventricular filling when he defined a dissociation between untwisting and filling, whereby 50–60% of recoil occurs during this isovolumic interval [61]. He reasoned that this motion may promote the suction that explains the explosive LV filling that follows the falling LV pressure below left atrial pressure, and wondered if unwinding released the potential energy stored in elastic elements during prior systolic deformation [61].

**Figure 10:**
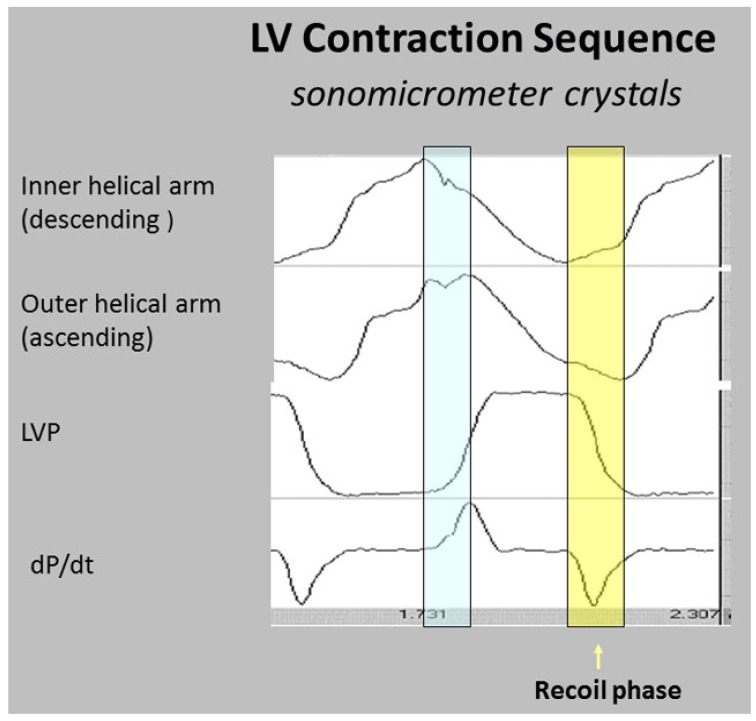
Left ventricular contraction sequence by anterior LV sonomicrometer crystals. Blue shading shows pre ejection isovolumic interval, yellow shading shows post ejection recoil. Note (a) ascending helical arm does not contract during pre-ejection, but contracts during recoil; (b) negative dP/dt is yardstick for starting recoil, and marks when inner helical arm stops contracting. Reproduced form the reference [6] with Publisher’s permission.

The interdependence of torsion and recoiling is a vital interaction because torsion must stop before unwinding can start. The post ejection isovolumic time frame interval is ~90 ms (Figure 10), but diastolic dysfunction may develop when protracted inner descending helix contraction widens this timing gap to >100 ms as seen during aortic stenosis, hypertension, and ischemia [58,62,63,64].

Current thoughts about recoil during isovolumic relaxation imply that the epicardium governs the clockwise rotation of the LV apex, suggesting that it reverses torsion’s apical counterclockwise rotation [61,65]. The dynamics of functional anatomy challenges this conclusion, since the epicardium (outer ascending helical muscle) is still contracting (Figure 10). Instead, the circumferential wrap causes clockwise rotation, as outlined previously for mitral valve opening. The term ‘isovolumic relaxation interval’ is incorrect because the ascending helix is still contracting, it should be renamed the ‘post ejection isovolumic period’.

This revision has historic precedence, as Wiggers can now join Harvey (who described compression versus twisting) and Vesalius (who described apex moving toward the base versus its natural base to apex motion) as being astounding titans that were not perfect.

## 6. Twisting or Torsion

There is agreement that the spiral architecture of LV muscle fibers produces LV systolic wringing or twisting during ejection by producing differential rotation of the LV base (clockwise) and apex (counterclockwise). This is called torsion by expressing its rotational angle, along the LV longitudinal axis. The responsible muscular mechanisms have important functional implications, yet these have only been deduced.

**Figure 11:**
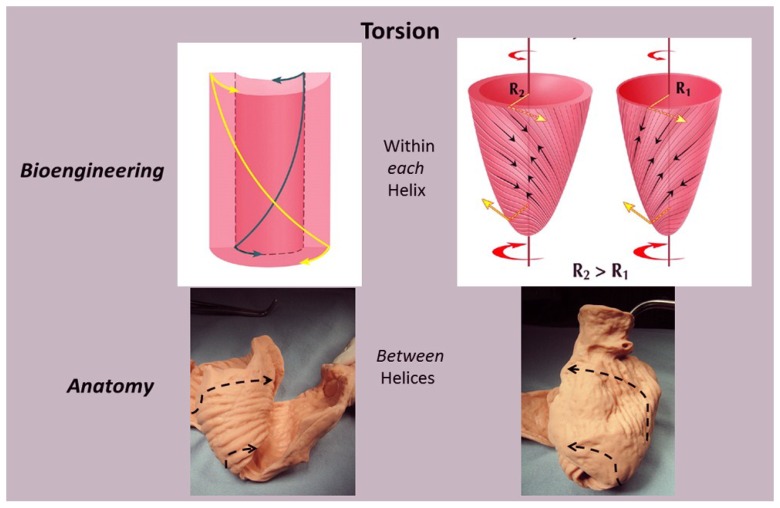
Torsion. The bioengineering approach (upper left) shows it develops ‘within each helix’; epicardial (outer) muscle has counterclockwise apex and clockwise base rotation, while endocardial (inner) muscle has clockwise apex and counterclockwise base rotation to reflect these reciprocal actions in each helical arm. The right image shows the suggested inner and outer cones occupied by the inner and outer helix. The lower images are anatomic: torsion develops ‘between helices’ as the entire inner descending helix rotates clockwise, and entire outer ascending helix rotates counterclockwise. Reproduced form the reference [8] with Publisher’s permission.

Taber’s bioengineering model has been accepted [66], as it introduces a helical architecture containing a single layer composed of obliquely aligned muscle fibers embedded in an isotropic matrix [65]. Torsion is described as being developed “within each helix arm”—epicardial fiber contraction rotates the apex counterclockwise and base clockwise, whereas subendocardial fiber contraction rotates them in the opposite directions. Geometric mechanisms differ, as torsion develops “between the helix arms” with the entire inner descending arm rotating the base clockwise, and the entire outer ascending arm rotating the apex counterclockwise (Figure 11).

Evidence for this structural explanation is supported by architectural and imaging studies. The anatomically unfolded heart reflects an uncoiled rope (Figure 1) that, when re-folded, contains a helix composed of the 60° overlapping of inner and outer spiral coils that aim at either the apex (inner helical arm) or base (outer helical arm). Septum strain recordings confirm deformation in different longitudinal directions [12,38], and high frequency echo tracings record the smooth transition of functionally overlapping fibers (Supplementary Video S2) which pass along or across its mid septal echogenic line [12].

Downward shortening of both helical coils cannot cause torsion, because the systolic septum longitudinal strain shows the positive deformation (elongation) of the outer ascending helical arm (Figure 4)—a motion that continues during recoil, evident by sono micrometer (Figure 10) and by MRI evaluation (Supplementary Video S3). Interactions between torsion and recoil are independent of torsion’s peak value, because prolonged torsion introduces a twist-based retardation of the unwinding required to develop suction.

Torsion and recoil affect ventricular performance in a way that relates to the contractile patterns of the inner descending and outer ascending helical arms. Torsion ends when the inner descending helical arm stops contracting—since uncoiling cannot begin until that happens. This recoil interval includes the post ejection isovolumic phase and first-third of diastole [67]. Prolonged torsion delays the recoil’s starting—becoming a precursor for development of diastolic dysfunction. This interdependence between torsion and recoil emphasizes why diastolic dysfunction cannot be thought to exist in a patient whose ejection fraction is considered normal and healthy.

## 7. Untwisting

Untwisting during recoil represents the antonym for twisting during ejection. This traditional term is used to describe the clockwise rotation of the apex that counteracts its counter-clockwise motion during twisting for ejection [55]. Yet the physical designations of these actions differ; “twisting” mirrors rotating the upper fist clockwise and the lower one counterclockwise, to confirm reciprocal left and right handed helical muscle rotations shown by MRI and echocardiogram. However, the MRI does not record the expected ‘untwisting counterpoint’ of apical clockwise and basal counterclockwise rotation. Instead, a global clockwise rotation exists during recoil [48]. It is caused by a dominant circumferential wrap that overpowers the outer ascending helical arm’s ongoing systolic counterclockwise motion.

Each muscle must return to its starting point, so these anatomic reasons do not counteract the recoil that helix and wrap muscles must develop. Instead, functional aspects of the HVMB explain why recoil’s expected global clockwise and counterclockwise ‘untwisting’ cannot occur. The term ‘untwisting’ should be changed into ‘uncoiling, recoiling, or unwinding’ to capture recoil’s inception point since it may evolve future diastolic dysfunction treatments.

## 8. Longitudinal and Circumferential Strain

Strain measurements define muscle deformation relative to original length by addressing “shortening in circumferential or longitudinal dimensions or thickening in radial dimensions”, but they do not define why deformation happens or what it means. For example, two-thirds of strain is circumferential and only one-third longitudinal, so that it is considered a more robust tool [40,68], especially because some believe the predominant muscle fiber orientation is followed [69].

The functional importance of deformation is linked to its muscular cause. Longitudinal strain reflects coiling downward of the oblique helical spiral fibers to generate the normal ~60% ejection fraction [13]. Conversely, short axis shortening arises from predominantly circumferential fiber deformation, but only a ~30% ejection fraction is yielded [13]. These fiber angulation changes have powerful implications, because impaired systolic contractile strength develops when the ventricle becomes spherical and the helical muscle fibers develop a more transverse orientation [13].

Longitudinal strain quantifies ventricular shortening, and was thought to reflect how the apical part pulls the ventricular base downward [70]. The helix and wrap architectural configuration application defines a different sequence, since longitudinal strain reflects how the spiral coil of the inner descending helical arm sequentially shortens due to its base-to-apex contraction—a trajectory that follows the human excitation studies showing that upper septum activation precedes apical stimulation. [71,72,73]. Longitudinal muscles “for pulling” do not exist in the ventricle (Figure 1) (except for thin papillary muscles), so that “pulling down” deductions contradict natural motion dynamics [70].

Two factors explain why circumferential deformation produces ventricular compression or cardiac narrowing. The first is short axis shortening of the wrap or basal loop’s transverse fibers. The second is transverse shortening produced by thickening of the contracting inner helix’s descending arm. Their individual contributions cannot be determined because they are superimposed within the LV free wall. Conversely, the septum does not contain a circumferential wrap so that its longitudinal strain measurement is possible (Figure 4).

Longitudinal strain reflects deformation of helical spiral fibers, and is measured by recording mitral annulus excursion toward the apex (or MAPSE mitral annular plane systolic excursion) [74]. Figure 6 shows the dominance of the spiral inner descending helical segment in producing longitudinal strain during systole.

**Figure 12:**
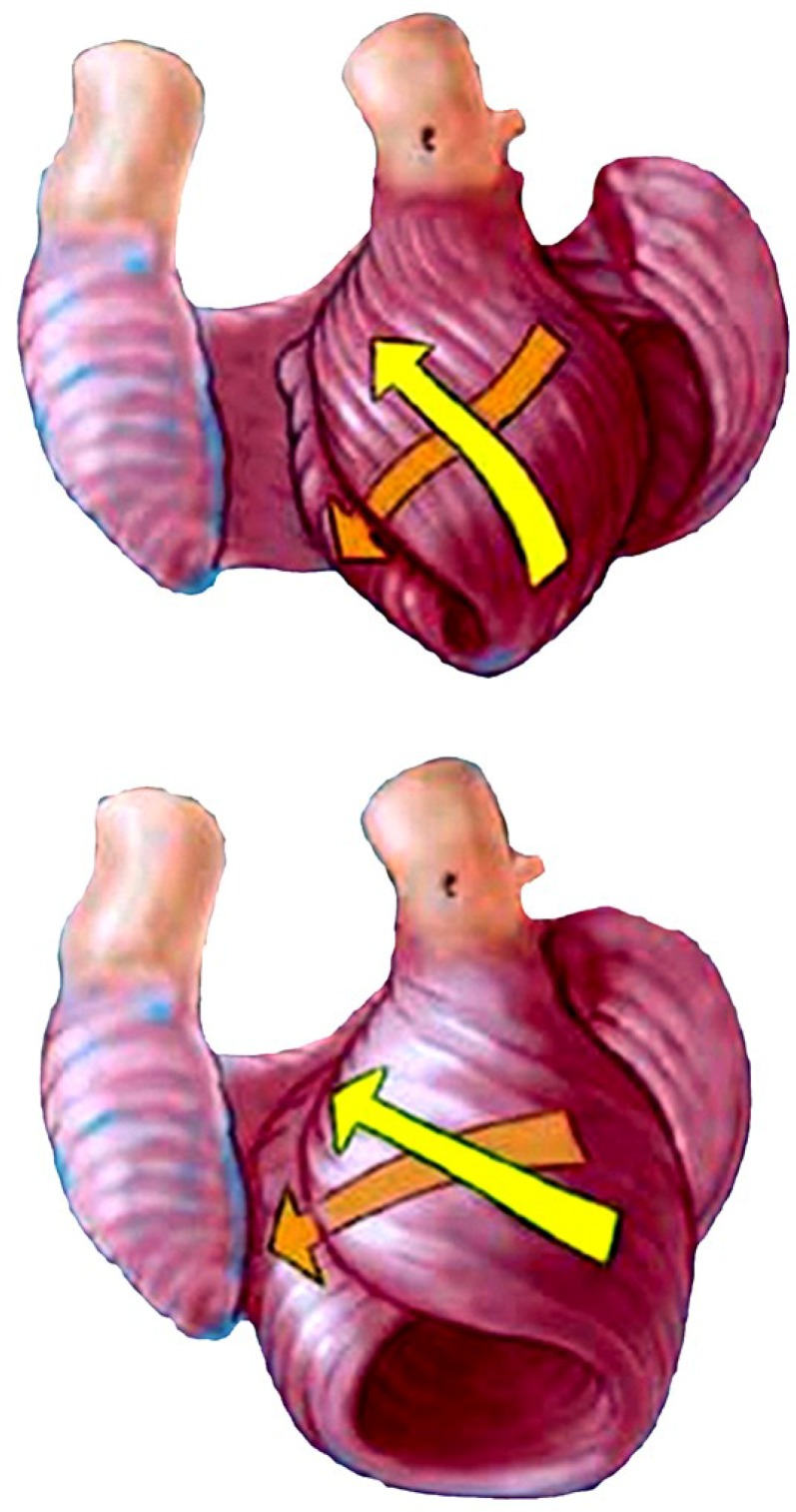
Helical fiber orientation (yellow arrows) in normal ventricle (above) with reciprocal 60° angulation and a conical shape. Spherical heart (below) shows a more transverse pattern with 45° or less angulation, mirroring the failing dilated heart. Reproduced form the reference [56] with Publisher’s permission.

Impaired longitudinal strain is an early finding in dilated hearts [75] and develops when the ventricular shape becomes spherical. This geometric change makes the natural oblique fibers develop a more transverse configuration [75,76] that disrupts their twisting capacity (Figure 12). This concept’s functional validity is confirmed by the consistent return the cardiac twisting when the failing heart’s spherical shape becomes rebuilt into its natural elliptical shape [77].

The surrounding basal loop fibers provide a short axis supporting restraint [78] that follows ventricular longitudinal strain limitation. For example, circumferential strain is augmented when intraoperative septum damage causes RV longitudinal systolic dysfunction [79]; the LV follows a similar sequence [69]. Conversely, increased ventricular sphericity follows loss of this circumferential restraint [78].

Worsened longitudinal systolic strain exists when relaxation is altered during diastolic dysfunction [80]. The query “is it only diastolic dysfunction?” arose after this coincidence was observed in hypertensive patients with diastolic dysfunction [81]. The differential function of the inner descending and outer ascending helical arms answers this mechanical question [58], as described in the upcoming section on diastolic dysfunction.

## 9. Regional Function versus HVMB

Regional echo based structure function analysis is linked to perfusion related changes [82]. Regional deformation (grading shortening and thickening) is aligned with the perfusion territories of 17 echo segments that are based within three circular rings of the basal, mid, and apical ventricle [82]. This arbitrary selection of transmural muscle (with comparable mass) into echo based LV sub-segmentation provides useful information about regional perfusion, yet except for segment 17 that shows absent apical deformation, this topographical approach does not record the performance dynamics forthcoming from HVMB analysis [45].

A similar arbitrary concept guides how readers are taught to understand the architecture of the helical muscles in the LV elliptical shape. They are considered to exist within overlapping cones, with “state of the art” reports [65] showing the inner cone describes endocardial muscle, and outer cone identifying epicardial muscle [65,83].

**Figure 13:**
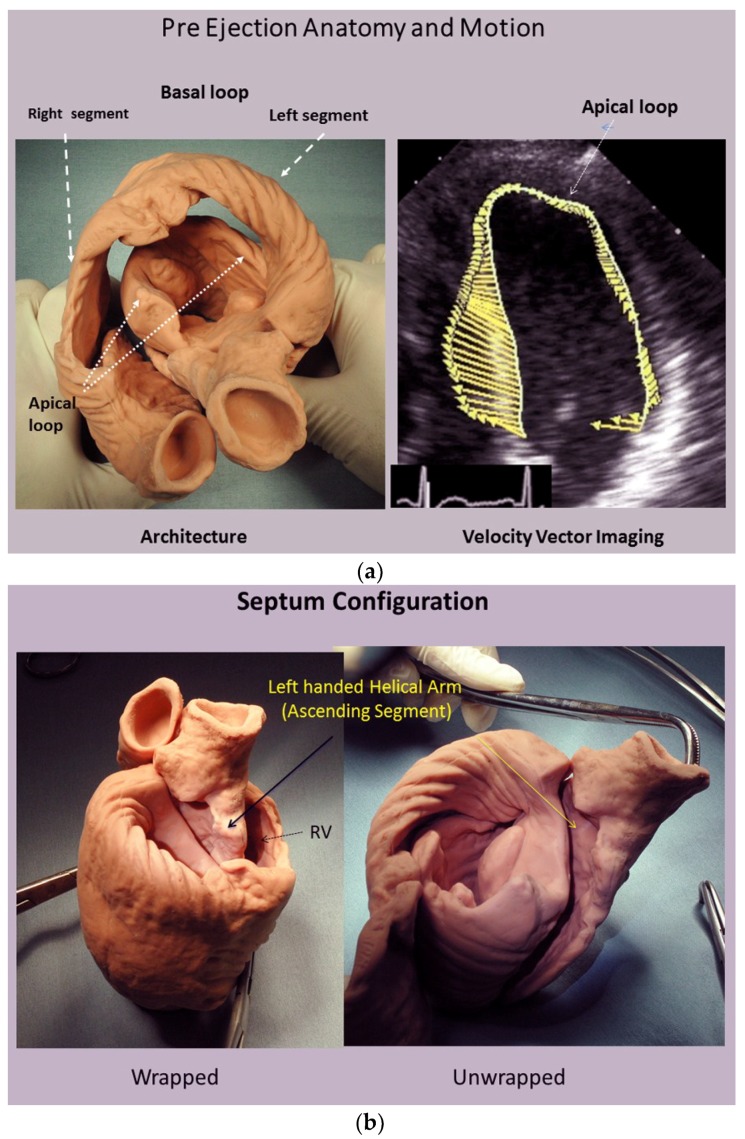
(**a**) Pre wall ejection motion from velocity vector imaging (VVI) is correlated with cardiac anatomy. The upper septum bulges into the right ventricle (upper right); (**b**) The helical architecture demonstrates absence of helical overlap in region beneath the aortic valve. Its endocardium is formed by the outer, ascending left handed helical arm that does not contract during this interval. Reproduced form the reference [8] with Publisher’s permission.

This designation is incorrect because the outer ascending helical arm forms different parts of the endocardium. An example of this unsuitability is that helical muscle overlap is absent in the septum muscle endocardium below the aortic valve [7]. Velocity vector imaging studies confirm this during the pre-ejection isovolumic phase, since the upper septum bulges like an aneurysm because contraction of outer ascending helix has not yet started [7,12] (Figure 13). The outer ascending helix also forms the endocardium in the posterior LV wall where helical overlap is absent. Validity of descriptions of the heart’s architecture is only possible by following the sheaths within muscle planes of its helical arms and circumferential wrap.

## 10. Resynchronization

Excitation contraction coupling provides the infrastructure behind how twist develops during each HVMB contraction. This process requires the natural flow of electrical impulse from the conduction system into the responsible myocytes. Insight into the HVMB anatomy and interventricular conduction relationship is gained by reviewing the evolution of clinical ‘resynchronization’ therapy (CRT). ‘Synchrony’ is the traditional term used to characterize the coordinated contraction of the LV, RV and septum—observed from two-dimensional echo or ventriculography. They record harmonized “all at once“ movement—mimicking the making of a fist with all fingers squeezing in unison in patients with a normal narrow QRS interval. Conversely, a wide QRS interval defines delayed regional electrical activation, and is associated with uncoordinated contraction that may produce septum bulging or billowing, mitral regurgitation, and raise LV volume.

‘Resynchronization’ is achieved by simultaneously pacing the LV and RV with the aim of restoring ‘synchrony’, but its portended treatment goals are only sometimes met [84]. For example, ~30% of patients do not respond [84], LV volume is not reduced in 40% [85], recovery of twisting is inconsistent [83,86], and late survival [87] increases only 0.85 years in comparison to optimal pharmacologic therapy. A suggested way to solve this dilemma is to search for a better site for LV pacing [85], but the validity of the ‘resynchronization concept’ must also be examined.

This change in thinking may be important because normal cardiac motion is not ‘synchronous’. Instead it is a ‘sequential’ motion that evolves when the electrical impulse traverses the natural conduction system and its collagenous cardiac netting matrix to stimulate individual muscle fibers. The clenched fist that is traditionally used to define contractility does not reflect an ‘all at once’ motion. Instead, it represents the ‘chronological closing’ of the little finger, ring finger, middle, and then index fingers, as they create the whirling motion which mirrors the normal heart’s natural twisting movement. Such coordinated torsion develops because the electrical propagation velocity is 10× faster (at 3 m/s) through the natural His-Purkinje fiber conduction system, than via direct ventricular muscle stimulation (at 0.3 m/s) [88]. Wiggers in 1925 described the functional dilemma of asynergic ventricular muscle motions that follow synchronous single electrical excitation [89]. His observation had no impact, because this form of stimulation persists to remain the unchanged stalwart of conventional pacing.

Twisting was recently evaluated by recording HVMB motions following either isolated (direct) ventricular, or biventricular stimulation (CRT), and torsion was inconsistent [90]. Conversely, pacing of the His-Purkinje system [91] returns the sequential activation responsible for twisting to restore natural torsion. The clash between these pacing avenues highlights that a limitation of CRT is that it reflects the two-dimensional approach of isolated site excitation which can only gain compression. In contrast, natural conduction excites the inbuilt conduction circuits which unfold the three-dimensional approach that returns cardiac twisting.

CRT provides mechanical, but not physiological improvements. For example, a wide QRS interval delays septum activation, so that the earlier LV free wall contraction will make it billow or bulge. The resultant ventricular stretching will tether the posterior papillary muscle (adjacent to septum) connected to the mitral leaflet, and produce mitral regurgitation from poor leaflet coaptation. CRT returns the septum to its midline position to offset papillary muscle tethering and remedy mitral regurgitation—but it does not consistently restore cardiac twisting. Conversely, His-Purkinje pacing rebuilds normal conduction by shortening the QRS interval [92]. It generates a sequential heart beat that permits the natural twisting motion to resume [93]. Awareness of these differences may hasten interest in evolving of His-bundle pacing approaches.

## 11. Right Ventricular Function

Right ventricular failure is difficult to manage because its underlying mechanisms are uncertain. Decision dilemmas follow such incomplete functional knowledge. For example, the RV is considered a passive chamber because early functional recovery follows its exclusion by the Glenn (superior vena cava to pulmonary artery) or Fontan (pulmonary artery to right atrium) procedures [94,95]. Yet this conclusion is contradicted by the ~40% mortality that develops in patients that occlude a right coronary artery containing large septal branches [96].

**Figure 14:**
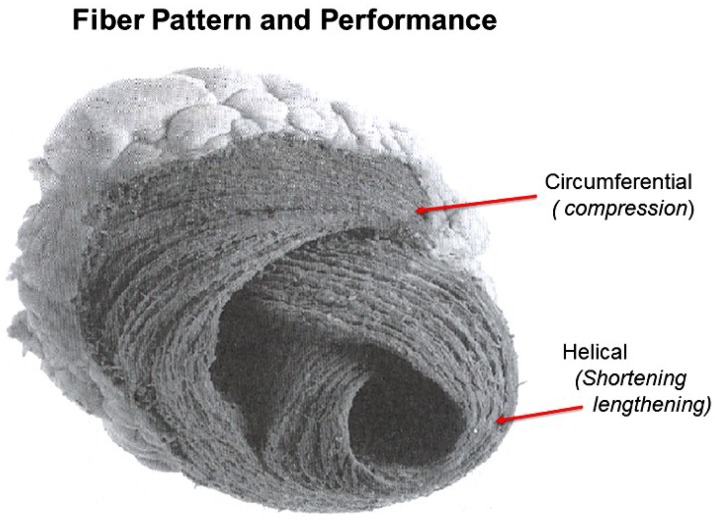
Right ventricular fiber pattern and HVMB, where the circumferential wrap or basal loop causes compression and narrowing, and the underlying helix with oblique fibers at 60° angles causes shortening and lengthening. Reproduced form the reference [8] with Publisher’s permission.

RV cardiac function is determined by myocardial fiber orientation (Figure 14). A thin circumferential wrap containing predominantly transverse muscle fibers forms its free wall. Their contraction produces compression via a bellows action that accounts for 20–30% of ejection fraction [13,97]. Conversely, only helical fibers construct its thicker midwall septum, and these oblique fibers [98] generate the twisting action responsible for 80% of RV global function, making the septum “the functional lion of the RV” [25,99].

Investigative studies document the dynamics of RV’s compression and twisting movements. For example, unaltered RV function follows RV free wall exclusion by cauterization, patch replacement [100,101] or its regional ventricular fibrillation [102] if the septum is intact. Yet RV failure follows septal injury when pulmonary hypertension co-exists [103]. This functional distinction between helix and wrap has not influenced the guidelines of the American Society of Echocardiography and European Association of CV Imaging that recommend measuring 2D STE-RV free wall strain, but do not identify that it accounts for only 30% of RV performance [82]. This functional discrepancy explains outcomes following a report that limited RV free wall ventriculotomy, aiming to avoid late RV failure [104]. It was not successful because of RV free wall’s minor effect on right heart performance.

The interplay becomes especially clear following cardiac surgery, where postoperative RV performance remains unimpaired, despite almost 50% of patients developing paradoxical septum motion [105]. But this compensatory circumferential wrap compression can only be effective when pulmonary vascular resistance (PVR) is low; RV failure develops if septum twisting is lost when PVR is increased [103]. This explains why the Glenn and Fontan procedures are contraindicated if PVR is high, since they exclude the septum, whose role is to the generate the twisting needed to counteract high afterload.

Tricuspid annular plane systolic excursion (TAPSE) quantifies the extent and rate of helically induced longitudinal strain [25] by documenting how ventricular shortening by the coiling helix will bring the base closer to the apex. The RV and LV chambers are thought to be topographically separate, but they share a common HVMB architecture, so that the twisting behind TAPSE in the RV reflects MAPSE in the LV. Vascular resistance is the counterforce to twisting and influences decision-making when right heart failure is caused by impaired septum function. If pulmonary pressure is high, treatment with vasodilator drugs (amrinone, milrinone) may be preferable to vasoconstrictor agents (epinephrine, dopamine) that would accentuate the afterload confronting the poorly contracting septum.

The interface between RV helical and circumferential muscle interaction is further clarified from RV function examination following either conventional or catheter based aortic valve replacement (AVR). Longitudinal and circumferential strain is unaltered in catheter based AVR that does not use cardioplegia. In contrast, the use of cardioplegia during surgical SVR results in the commonplace finding of septal damage [105]. This injury had an associated 50% reduction in TAPSE (the helix), which resulted in a 60% increase of compensatory circumferential strain (the wrap) [79].

RV failure sometimes develops in heart failure patients with high PVR following implantation of a left ventricular assist device (LVAD). Ventricular decompression by LVAD changes cardiac geometry by collapsing the LV. This maneuver bows the septum toward the left side, making its helical fibers more transverse and dysfunctional [56]. RV failure ensues, but can be quickly resolved by diminishing the extent LV decompression in order to mechanically restore the septum’s midline position as it re-establishes its natural geometry [106].

## 12. Diastolic Dysfunction

Diastolic dysfunction is characterized by how impaired ventricular relaxation and increased wall stiffness can limit LV filling in ~50% of patients with heart failure, despite their having normal ejection fraction [107]. Nishimura’s [67] classic 1997 overview emphasized empirical treatment, because of absent clear-cut pathophysiologic concepts—a limitation that still prevails. Historian David McCullough observed a similar conundrum in politics, stating “it is like trying to repair an engine that you do not know how to take apart”.

Normal ventricles receive 70% of their filling during the first third of diastole, but uncertainty exists about why this happens. Uncoiling via apical clockwise rotation influences early filling, but this motion begins in the last half of systole and extends to the first third of diastole [67]. Despite its acknowledgment [67,108], calling this interval “passive” rapid ventricular filling introduces a vis a tergo mechanism with reliance upon an intraventricular pressure gradient (IVPG) that promotes blood movement toward the apex [108,109].

The alternate mechanism is “active” ventricular suction to aspirate atrial blood, using peak negative dP/dt and the time constant of relaxation (tau) as measurement indexes [67]. Understanding the energy conversion process needed for either active recoil [110] or via a muscular mechanism [109,111] has been its missing component. The HVMB structure function relationship identifies this answer.

There is clear-cut understanding of how ventricular pressure increases during filling of a stiff ventricular wall, yet this “compliance mechanism” is absent during normal rapid ventricular filling, where ventricular pressure falls as volume increases [112]. “Diastolic suction” explains this process, as it draws ventricular blood from the atrial reservoir [113,114,115,116] to create a negative diastolic pressure. Brecher documented this negativity from recordings of the heart withdrawing blood from a lower reservoir [114].

The current term “passive rapid ventricular filling” parallels the prior controversy between twisting or compression for ejection. Background exists, as Galen, in 180 AD described “the overlying heart, at each diastole, robs the vena cava by violence of a quantity of blood”. Wiggers, in 1921, rejected a vis a fronte ventricular filling [117], and Roberts, in 1979, described “suction” by finding the left atrium had invaginated into the left ventricle through the mitral valve [118] during a post mortem exam after terminal hypovolemic hemorrhage.

Concepts about elastic recoil or systolic ventricular filling are best tested by evaluating the integration of form and performance. Van Dalen’s elegant series of observations [26,119,120,121,122] clarified the echo based dynamics of “untwisting”, but he ascribed these motions to oblique muscles. He reasoned that repolarized epicardial fibers (outer helical arm) actively untwisted to move clockwise, since endocardial fibers (inner helical arm) were reported to be still depolarized [50]. His focus upon “untwisting” as a new diastolic dysfunction treatment goal is an important consideration (as described below), but his suggested mechanism cannot occur. The helix is not involved in post ejection isovolumic phase recoil, because unwinding is caused by the basal loop whose contraction has stopped [6]. Conversely, the ascending segment (epicardium) is still contracting (depolarized) during the isovolumic interval, so that its persistent counter-clockwise motion would oppose the prevailing clockwise movement (Figure 10) [6,33,41].

Torrent Guasp’s geometric contributions are central to our structure/function approach, but his “systolic ventricular filling” concept [109] uses only form to deduce function. He ascribes it to ongoing contraction of the ascending (outer helical) arm. HVMB dynamic analysis certifies that its post ejection isovolumic phase shortening has stopped before rapid filling begins (Figure 10). Consequently, its elastic recoil causes the explosive ventricular filling that follows its springing back into its starting position. As stated, uncoiling may reflect how stored potential energy during ejection [61] is released during this unwinding process that creates a centrifugal force for aspiration of atrial blood.

The interdependence of torsion and recoil are fundamental concepts behind understanding how HVMB clarifies why diastolic dysfunction develops. The central theme is that recoil cannot start until torsion ends. The recoil process causes the predominance of ventricular filling in the healthy heart, yet 50% of this unwinding exists during the pre-filling phase, reaching 60% after catecholamine infusion [61,123]. The remainder of recoil happens during rapid filling and is due to uncoiling of the outer ascending helical arm [6].

The time-frame between the end of contraction of the inner descending and then, of the outer ascending helix arms, creates a “temporal hiatus” (Figure 10), and this “time gap” becomes the centerpiece for understanding the HVMB muscular actions that are responsible for diastolic dysfunction. Curtailment of recoil will restrict suction and by narrowing of this temporal hiatus. Consequently, a vis a tergo mechanism must be used to cause ventricular filling by raising the compensatory factor of atrial pressure. Pressure related enhancement of filling is normal after atrial contraction at the end of diastole, but lung congestion may follow its presence during early diastole.

Disruption of the HVMB dynamics responsible for the interface between torsion and recoil creates diastolic dysfunction in several ways, because prolonged systole during torsion will encroach upon the aforementioned “temporal hiatus” during aortic stenosis [62], hypertrophic cardiomyopathy, ischemia [64], impaired sarcolemmal calcium flux efficiency [124], and age-related calcium turnover [124]. Its genesis is that unwinding cannot start until the prolonged inner descending helix arm contraction has ended.

Most importantly, thoughts of diastolic dysfunction that focus upon an isolated relaxation disorder must be reexamined, since each patient shows combined systolic and diastolic abnormalities [125]. These alterations involve impaired ventricular twisting and longitudinal deformation (strain) patterns [81] that delay untwisting to reduce suction and impair early diastolic filling [126]. Restricted helical systolic function is evident by reduced longitudinal strain, despite normal ejection fraction. This corresponds to how the prolonged torsion in patients with aortic stenosis can compromise their uncoiling process [62].

Mechanism related treatments can reverse diastolic dysfunction, since aortic valve replacement allows regression of LV hypertrophy with resulting return of twisting and recoil to normal [127]. Post ischemic diastolic dysfunction is reversed when sodium hydrogen ion inhibitors limit calcium accumulation within the inner helix. Their avoidance of prolonged contraction restores the natural time gap between the end of inner and then outer helix shortening to allow the recovery of the suction that accentuates ventricular filling [58].

**Figure 15:**
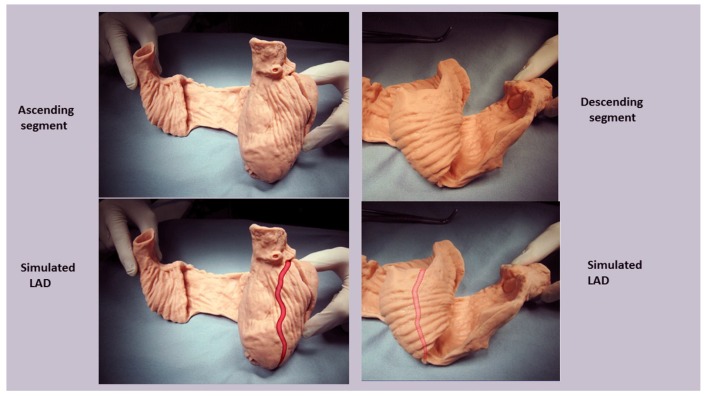
Figure 1 anatomy modification with “simulated left anterior descending artery” that is a vascular highway that bisects the helix, which constructs the septum and the LV free wall. Anatomy (above), and how (below) LV free wall is on vessel’s left side, and septum (which is three-dimensionally deeper) is on right side.

The LV free wall and septum are formed by the same HVMB muscle mass (Figure 1 and Figure 15) and this association confirms why diastolic dysfunction after routine cardiac surgery is clinically important. Diastolic and septum dysfunction are similar, but this conclusion has not yet been appreciated. It is well known that septum dysfunction is commonplace after cardiac surgery, as 43% of 3292 patients [105] develop septal paradoxical motion (lesser damage was not reported). Impaired myocardial protection causes this injury, but this damage can be avoided [128], as undamaged hearts show improved longitudinal helical deformation [129].

Diastolic dysfunction, its sequel, develops in 44% to 75% of patients undergoing coronary grafting or aortic valve procedures [130,131,132]. This incidence mirrors the frequency of septum damage [105] and both entities may disappear in 6–12 months [133,134]. Conventional consideration has not recognized the similarity between septum damage and diastolic dysfunction, because heart anatomy had been viewed topographically (LV, RV, septum), yet their coincidence is apparent and becomes predictable when the HVMB is used to recognize their commonality.

## 13. Conclusions

This rethinking of the core cardiology values started after learning of the contributions of Francisco Torrent Guasp. His studies exposed the simple design of cardiac architecture by showing that the HVMB contains a helix and a circumferential wrap. These two structures create the functional mechanics behind cardiac motion and thus define ‘what the heart is’. Application of this foothold knowledge reveals that many ‘accepted’ cardiac events are based upon deductions drawn from presumed myocardial structure, rather than from its natural configuration. Torrent Guasp’s revolutionary contribution opens the door toward an exciting future for the understanding, diagnosis, and treatment of cardiac disease.

## Supplementary Materials

The following are available online:

**Video S1** (http://www.mdpi.com/2308-3425/5/2/33/s1): Francisco Torrent Guasp doing cardiac dissection to unravel the HVMB;

**Video S2** (http://www.mdpi.com/2308-3425/5/2/33/s2): MRI of Mitral Valve Opening. Note MVO occurs while the aortic valve is open, and its inception is just as the septum begins to elongate. Recoil begins as the mitral valve opens, an event that accompanies the beginning of clockwise ventricular rotation evident on next MRI video; Reproduced form the reference [6] with Publisher’s permission;

**Video S3** (http://www.mdpi.com/2308-3425/5/2/33/s3): Short axis apex MRI that displays cardiac rotation and papillary muscle motion. Note the counterclockwise rotation during torsion, and closeness of papillary muscles. Clockwise motion is associated with a widening or the distance between papillary muscles. They attach to the mitral leaflets and leaflet separation (mitral opening) accompanies the clockwise ventricular motion during recoil;

**Video S4** (http://www.mdpi.com/2308-3425/5/2/33/s4): Short axis high resolution echocardiographic view of torsion and recoil in normal heart. The inner and outer mobile arms correspond to how they are portrayed in the Figure 5 post mortem MRI images. Note (a) the smoothness of these superimposed reciprocal movements, as the inner helical arm rotates clockwise, and outer helical arm rotates counterclockwise during torsion development and (b) how the inner and outer coils spring back to their starting position during recoil.
